# Assessment of Preparedness for Remote Teaching and Learning to Transform Health Professions Education in Sub-Saharan Africa in Response to the COVID-19 Pandemic: Protocol for a Mixed Methods Study With a Case Study Approach

**DOI:** 10.2196/28905

**Published:** 2021-07-28

**Authors:** Mike Nantamu Kagawa, Shalote Chipamaunga, Detlef Prozesky, Elliot Kafumukache, Rudo Gwini, Gwendoline Kandawasvika, Patricia Katowa-Mukwato, Rangarirai Masanganise, Louise Pretorius, Quenton Wessels, Kefalotse S Dithole, Clemence Marimo, Aloysius Gonzaga Mubuuke, Scovia Nalugo Mbalinda, Lynette van der Merwe, Champion N Nyoni

**Affiliations:** 1 Department of Obstetrics and Gynaecology School of Medicine Makerere University College of Health Sciences Kampala Uganda; 2 Faculty of Medicine and Health Sciences University of Zimbabwe Harare Zimbabwe; 3 Faculty of Medicine University of Botswana Gaborone Botswana; 4 School of Nursing Sciences University of Zambia Lusaka Zambia; 5 National University of Science & Technology Bulawayo Zimbabwe; 6 Faculty of Health Sciences University of Namibia Windhoek Namibia; 7 Department of Research School of Medicine Cavendish University of Zambia Lusaka Zambia; 8 Department of Radiology School of Medicine, College of Health Sciences Makerere University Kampala Uganda; 9 Department of Nursing School of Health Science Makerere University College of Health Sciences Kampala Uganda; 10 Faculty of Health Sciences University of the Free State Bloemfontein South Africa

**Keywords:** Africa, COVID-19, emergency remote teaching, formal online learning, pandemic

## Abstract

**Background:**

The current COVID-19 pandemic is affecting all aspects of society worldwide. To combat the pandemic, measures such as face mask–wearing, hand-washing and -sanitizing, movement restrictions, and social distancing have been introduced. These measures have significantly disrupted education, particularly health professions education, which depends on student-patient contact for the development of clinical competence. The wide-ranging consequences of the pandemic are immense, and health professions education institutions in sub-Saharan Africa have not been spared.

**Objective:**

This paper describes a protocol for assessing the preparedness of selected health professions education institutions in sub-Saharan Africa for remote teaching and learning during the COVID-19 pandemic.

**Methods:**

A mixed-methods design with a case study approach will be used. The awareness, desire, knowledge, ability, and reinforcement model of change was selected as the conceptual framework to guide the study. Eight higher education institutions in 6 sub-Saharan countries have participated in this study. Data will be collected through electronic surveys from among whole populations of academic staff, students, and administrators in undergraduate medicine and nursing programs. Qualitative and quantitative data from each institution will be analyzed as a case study, which will yield an inventory of similar cases grouped for comparison. Quantitative data will be analyzed for each institution and then compared to determine associations among variables and differences among programs, institutions, or countries.

**Results:**

Our findings will provide information to higher education institutions, particularly those offering health professions education programs, in Africa regarding the preparedness for remote teaching and learning to influence efforts related to web-based teaching and learning, which is envisaged to become the new normal in the future.

**Conclusions:**

This study has not received any funding, and any costs involved were borne by individual consortium members at the various institutions. Ethics approval from the institutional review board was obtained at various times across the participating sites, which were free to commence data collection as soon as approval was obtained. Data collection was scheduled to begin on October 1, 2020, and end on February 28, 2021. As of this submission, data collection has been completed, and a total of 1099 participants have been enrolled. Data analysis has not yet commenced.

**International Registered Report Identifier (IRRID):**

DERR1-10.2196/28905

## Introduction

### Background

The World Health Organization declared COVID-19 a pandemic on March 11, 2020, and countries had to adopt containment and mitigation measures such as restrictions on movement of persons and human congregation [1,2]. Consequently, many educational events that required people to congregate were put on hold or canceled, and the governments of many African countries decided to close educational institutions in an effort to contain the disease outbreak. Health professions education was no exception to these drastic measures, and planned teaching and learning activities have been almost totally disrupted. For most institutions in Africa, COVID-19–related restrictions were too rapid to institute any well-planned contingency measures to ensure the continuity of teaching and learning activities. Many students experienced complete stoppage of planned teaching and learning activities for unknown timespans. Students and teachers were physically isolated and disconnected from the mainstream university setting, where face-to-face instruction was the dominant approach to teaching and learning. The COVID-19 pandemic arguably exposed the unpreparedness of many higher education institutions (HEIs) in Africa to alternative teaching and learning approaches [3].

Consequent to the COVID-19 pandemic, many HEIs adopted emergency remote teaching and web-based learning by using a blended approach with reduced physical presence of students and educators on campus [4]. For educators to continue engaging with their students even during the pandemic, creative approaches such as “emergency remote teaching” (ERT) or “formal online learning” (FOL) needed to be adopted for continued teaching and learning. ERT is described as a short-term solution aimed at providing temporary access to instruction in a manner that is rapid to set up and reliable during an emergency crisis such as the COVID-19 pandemic, while FOL is a formal system of web-based learning, which is well-established and takes months to prepare [5]. While suggestions for FOL have taken on an increased impetus, they are not new. The World Health Organization, in its recent publication [6], recommended FOL as a tool for interprofessional education, particularly if it is delivered in an open-access environment [6].

Remote teaching and learning, however, are not without their challenges even in high-income countries. A McKinsey and Company report [7] in May 2020 noted that in the United States, students’ ability to succeed in a remote learning environment was affected by differences in household income; less than half (40%) of students from low-income households reported being able to access the necessary equipment for remote learning compared to 72% of students from high-income households, while only 56% of students from low-income households reported having reliable internet access and only 45% reported that their home environment supported remote learning, compared to 77% and 64% of students from high-income households, respectively [7]. This pattern is unlikely to be different or even worse for students in most HEIs in Africa, where additional issues such as power supply, internet connectivity, sociocultural dynamics such as gender roles, educators’ and leaners’ competence related to remote teaching, and security may be of concern [8,9].

Literature on how HEIs, particularly those offering health professions education programs, have adopted FOL and ERT and remote learning during the COVID-19 pandemic is fast emerging. However, most of this literature is emerging from high-income countries that have better resources and different socioeconomic contexts from those of African countries. Experiences with FOL and ERT in resource-poor countries, including those in sub-Saharan Africa, have been less documented, even though students have somehow continued to engage in the learning process [10,11]. Although HEIs in Africa continue to struggle to fully embrace FOL [8,9], there have perhaps been efforts implemented to utilize platforms that are currently available to most students and teachers to ensure that learning material, assessments, and feedback reach the intended audience. One such effort may be the use of ERT as a foundation to the development of comprehensive web-based learning in the long term and it being considered the “new normal.” However, a question arises, which will guide this study: what progress has been made by HEIs in sub-Saharan Africa to transition to ERT or FOL within the current situation resulting from the COVID-19 pandemic?

The need to respond to the effects of the COVID-19 pandemic urgently may imply that institutions adopt web-based learning approaches, and that ERT presents an opportunity for web-based learning even in circumstances of limited internet connectivity [5]. We argue that ERT and remote learning is a possible foundation for future formal web-based teaching and learning strategies in most HEIs in Africa, and insights obtained from our experience with this approach in sub-Saharan Africa could influence the adoption of appropriate web-based teaching and learning models throughout Africa.

This study aims to describe the preparedness of selected health professions educational institutions in sub-Saharan Africa for remote teaching and learning during the COVID-19 pandemic. This paper describes a research protocol that was designed by a consortium of health professions education researchers from 8 HEIs in 6 sub-Saharan African countries.

### Conceptual Framework and Research Questions

**Figure 1:**
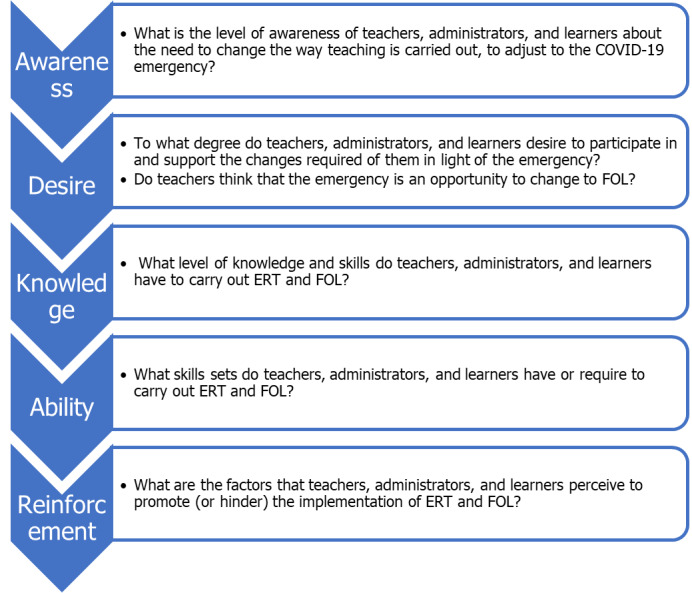
The conceptual framework of the study based on the awareness, desire, knowledge, ability, and reinforcement model [11]. ERT: emergency remote teaching, FOL: formal online learning.

The awareness, desire, knowledge, ability, and reinforcement (ADKAR) model of change was selected as the conceptual framework underpinning the study [12,13]. The ADKAR model focuses on change that is driven by the needs of the individual, whereby an evaluation is performed for the following attributes: awareness of the need for change, desire to participate in and support the change, knowledge necessary for change, ability or skills available or required to implement the change, and reinforcement to sustain the change. The use of this model will reveal factors that are critical for the success of FOL and remote teaching [14-16]. Figure 1 illustrates the ADKAR model that was used to generate the research questions to be answered in this study.

## Methods

### Ethical Considerations

Relevant safeguards required in studies involving human participants, as outlined in the Belmont report of 1979 and the International Conference on Harmonization (ICH) of 2002, will be applied as the overarching ethics framework for this study [17,18].

Ethics approval was obtained from the institutional review board at various timepoints across the participating sites, which were free to commence data collection as soon as ethics approval was obtained. Data collection was scheduled to begin on October 1, 2020, and end on February 28, 2021. As of this submission, data collection was concluded, and a total of 1099 participants were enrolled. Data analysis had not yet commenced.

### Study Design

The study will use a concurrent mixed-methods design. The ADKAR organizational change model will guide the approach to data collection, analysis, and reporting [13,14]. Data will be collected for 2 purposes: first, to construct case studies from each institution, which will provide an in-depth multifaceted insight into the preparedness of selected health professions education institutions in sub-Saharan Africa for remote teaching and learning during the COVID-19 pandemic and how they plan to conduct their training in the future [19]; and second, to compare quantitative data statistically by analyzing associations among variables and differences among programs, institutions, and countries.

### Study Setting

The study will be conducted at 8 health professions training institutions in 6 sub-Saharan African countries: University of Botswana, Botswana; University of Namibia, Namibia; University of the Free State, South Africa; Makerere University, Uganda; University of Zambia and Cavendish University, Zambia; and University of Zimbabwe and the National University of Science and Technology, Zimbabwe.

### Study Population and Sampling

The study population (Table 1) will consist of administrators, educators, and students from the medicine and nursing programs at the selected HEIs. For the total study population, purposive sampling will be used, which will target all the students, educators, and administrators at the aforementioned 8 HEIs. At each of the selected HEIs, whole populations of administrators and educators of undergraduate medical and nursing students before and during the COVID-19 pandemic will be invited to participate in the study. Whole populations of undergraduate medicine and nursing students will also be invited to participate in the study (N=9095).

**Table 1:** Study population.

Group	Medicine	Nursing	Total
Administrators, n	134	41	175
Educators, n	690	238	928
Students, n	4866	3126	7992
Total, n	5690	3405	9095

### Data Collection

Data will be collected using questionnaires with structured and semistructured elements (Multimedia Appendix 1). Instruments will be pretested by excluding 1 person in each sampled program from the sampled study population. This will assist in instrument validation. The questionnaires will be sent via the most practical route to all sampled participants. All data will be entered into an electronic database.

### Data Analysis and Presentation

Data analysis will be undertaken in stages. For each program, quantitative data will be summarized using a frequency distribution and averages, and qualitative data using thematic analysis.

In the first stage, a case study will be constructed for each of the 8 HEIs represented and will be presented discursively in paragraphs. Numbers will be used to indicate the strength of statements, and quotes shall be used to emphasize common issues. The case study framework will be based on the ADKAR model (Multimedia Appendix 1).

In the second stage, programs will be grouped in accordance with the extent to which they have planned for and implemented ERT and FOL. This will facilitate comparisons among categories of cases. Each institution will have the liberty to perform subgroup analysis based on data obtained from the institution.

In the third stage, quantitative data on each of the 5 ADKAR elements will be analyzed statistically to determine differences and associations among the programs. This will strengthen the understanding of the preparedness of selected health professions education institutions in sub-Saharan Africa for remote teaching and learning during the COVID-19 pandemic.

## Results

We hope to obtain detailed information regarding the ADKAR elements among the administrators, faculty, and students for remote teaching and learning during the COVID-19 pandemic at selected health professions education institutions in sub-Saharan Africa. This will be achieved in the form of case studies that will provide a detailed insight in to each program, institution, or country. Comparisons will be made, highlighting similarities, differences, and associations among the various ADKAR elements to further enhance our understanding of the level of preparedness by the various programs, institutions, or countries.

## Discussion

### Principal Findings

We propose to write a comprehensive report, which will be shared with program leaders at the various participating institutions. We hope that our findings provide information to HEIs, particularly those offering health professions educational programs, in Africa regarding their preparedness for remote teaching and learning. We believe that our findings will influence efforts related to web-based teaching and learning, which is envisaged to become the “new normal” in the future. We shall develop papers intended for submission to peer-reviewed journals to share our findings with a wider audience.

As of this submission, data collection was concluded, and a total of 1099 participants have been enrolled. Data analysis had not been commenced.

### Limitations

At each institution, only 2 of the many programs will be sampled: nursing and medicine. The information may not be fully representative of the level of preparedness of all the programs at these institutions. If response rates are low, statistical comparisons may not be possible or valid. This study involves a change in culture or tradition, which is quite complex since multiple factors are involved: environment, teachers’ attitude toward ERT and students’ ability to remain focused and self-motivated, parental support, and government support.

## Appendix

Multimedia Appendix 1 The case study framework.

## Abbreviations

ADKAR: awareness, desire, knowledge, ability, and reinforcement
ERT: emergency remote teaching
FOL: formal online learning
HEI: higher education institutions
