# Supplementary material for: Assessment of Preparedness for Remote Teaching and Learning to Transform Health Professions Education in Sub-Saharan Africa in Response to the COVID-19 Pandemic: Protocol for a Mixed Methods Study With a Case Study Approach
Source: JMIR Res Protoc. 2021 Jul 28;10(7):e28905. doi: 10.2196/28905 (PMC8320735; doi:10.2196/28905)
Supplement: Multimedia Appendix 1 [file resprot_v10i7e28905_app1.docx]

**Transforming health professions education in sub-Saharan Africa in response to the COVID-19 pandemic: an assessment of preparedness for remote teaching and learning – Research Protocol**

### **Appendix 1; The case study framework**

1. Name: the university, country and type of program

2. An overall statement of how the measures taken to combat the pandemic affected the program: initially (NOTE: one of the main issues that will help us group the case studies)

3. How students report being affected:

● How they used to work

● Their Awareness of the need for change

● Their Desire to participate in and support the change

● How they had to change their usual way of working

● Whether they had the Knowledge and the Ability to implement the change

● The factors that helped or hindered them to achieve and Reinforce the change: devices, connectivity, materials, skills, support, other

4. How teachers report being affected

● How they used to work

● Their Awareness of the need for change

● Their Desire to participate in and support the change

● How they had to change their usual way of working

● Whether they had the Knowledge and the Ability to implement the change

● The factors that helped or hindered them to achieve and Reinforce the change: devices, connectivity, materials, skills, support, other

5. How administrators report being affected

● How they used to work

● Their Awareness of the need for change

● Their Desire to participate in and support the change

● How they had to change their usual way of working

● Whether they had the Knowledge and the Ability to implement the change

● The factors that helped or hindered them to achieve and Reinforce the change: devices, connectivity, materials, skills, support, other

6. How well the program adapted overall and what happened subsequently as time went on (NOTE: one of the main issues that will help us group the case studies)
